# (2-Hy­droxy­phen­yl)(4,2′:4′,4′′-terpyridin-6′-yl)methanone

**DOI:** 10.1107/S2414314620008573

**Published:** 2020-07-10

**Authors:** S. Devika, Noor Shahina Begum, Kiran B. Manjappa, Ding-Yah Yang

**Affiliations:** a Department of Chemistry, Bangalore University, Jnana Bharathi Campus, Bangalore-560 056, Karnataka, India; b Graduate Program for Biomedical and Materials Science, Department of Chemistry, Tunghai University, No. 1727, Sec. 4, Taiwan Boulevard, Xitun District, Taichung-40704, Taiwan; University of Zürich, Switzerland

**Keywords:** crystal structure, terpyridine, ketone, crystal structure

## Abstract

The title compound, C_22_H_15_N_3_O_2_, can be described as a ketone with a phenol substituent and a terpyridine ligand coordinated to the carbonyl group. The three six-membered rings of the terpyridine ligand are not coplanar. The dihedral angles between the mean planes of the central ring and the external pyridine ligands are 22.77 (9) and 26.77 (7)°. The central ring of the terpyridine ligand is also not coplanar with the *o*-hy­droxy phenyl ring, the dihedral angle being 39.72 (5)°. An intra­molecular O—H⋯O hydrogen bond occurs. The crystal structure of the title compound is consolidated by C—H⋯O and C—H⋯N hydrogen bonding inter­actions.

## Abstract 2

The crystal structure of the title compound exhibits an intra­molecular O—H⋯O hydrogen bond and is consolidated by C—H⋯O and C—H⋯N hydrogen-bonding inter­actions.

## Graphical abstract

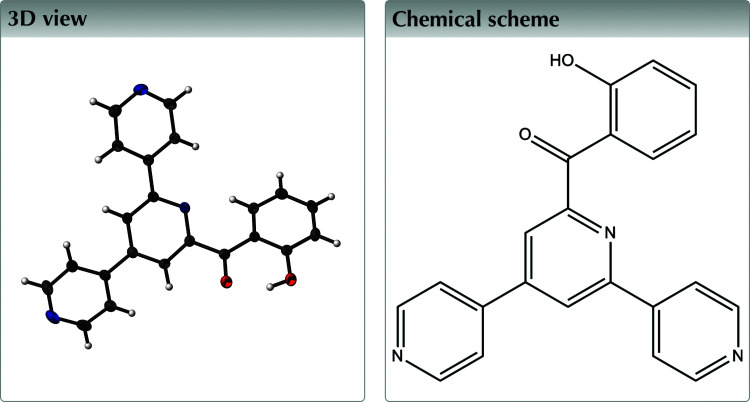

## Structure description

Pyridine and derivatives play critical roles in the biochemical field. The hydrazone derivatives of benzoyl­pyridines exhibit cytotoxic activity towards tumor cell lines (Santos *et al.*, 2018 ▸) and show excellent anti­proliferative activity (Kalinowski *et al.*, 2007 ▸). The quest for an efficient method for the preparation of such pyridine derivatives is ongoing. Herein, we report a novel method for the preparation of a 2-benzoyl­pyridine derivative in a one-pot reaction of 3-amino­coumarin with 4-acetyl­pyridine. The structural elucidation of the compound by spectroscopic investigation and single-crystal analysis has been performed.

**Figure 1:**
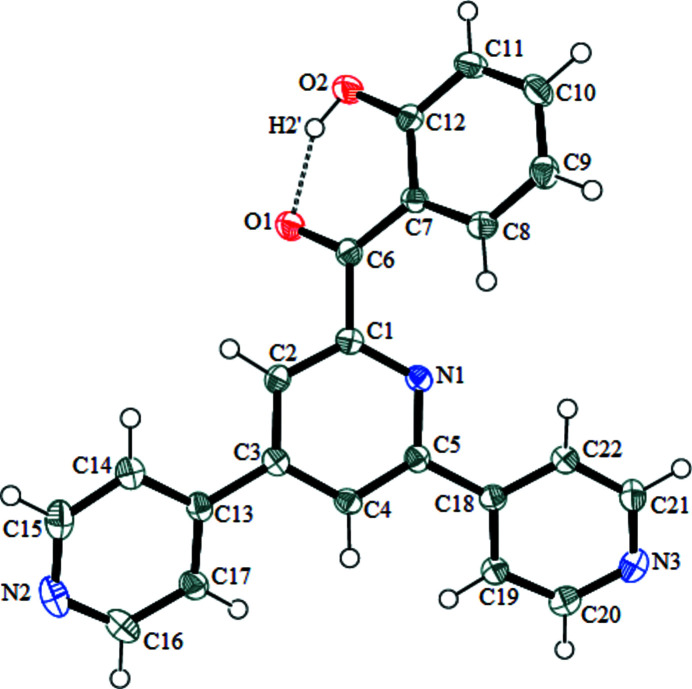
The mol­ecular structure of the title compound with the atom-numbering scheme. Displacement ellipsoids are drawn at the 50% probability level. H atoms are presented as small spheres of arbitrary radius.

The mol­ecular structure of the title compound is shown in Fig. 1 ▸. It crystallizes in the ortho­rhom­bic crystal system in *P*2_1_2_1_2_1_ space group with one mol­ecule in the asymmetric unit. The compound can be described as a ketone with a phenol substituent and a terpyridine ligand coordinated to the carbonyl group. The dihedral angle between the mean planes of the central ring of the terpyridine ligand and the *o*-hy­droxy phenyl ring is 39.72 (5)°. The three six-membered rings of the terpyridine ligand are not coplanar. The dihedral angles between the mean planes of the central ring and the external pyridine ligands are 22.77 (9) and 26.77 (7)° (for C13–C17/N2 and C18–C22/N3, respectively). A strong intra­molecular O—H⋯O hydrogen bond (O2—H2′⋯O1) exists between the O1 and O2 atoms of the phenol ring (Table 1 ▸).

**Figure 2:**
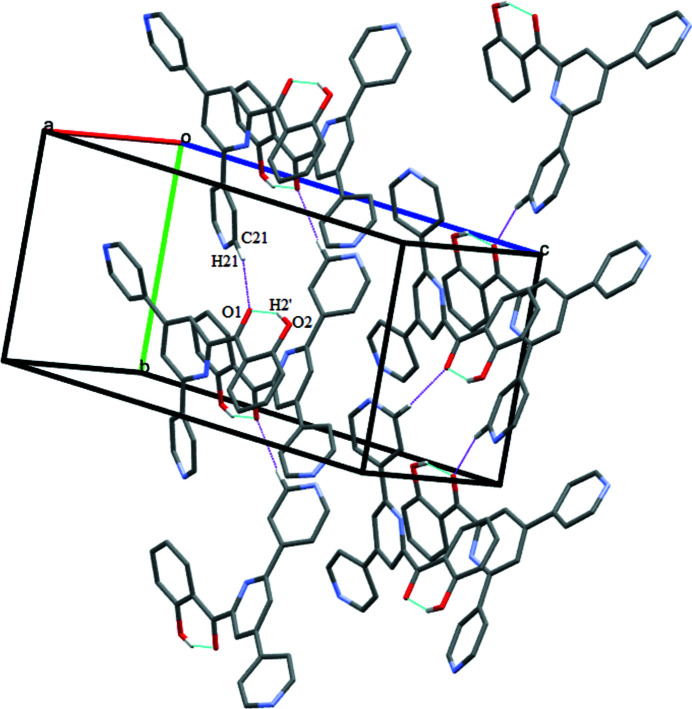
Unit-cell packing of the title compound showing intra­molecular O—H⋯O and inter­molecular C—H⋯O inter­actions as dotted lines. H atoms not involved in hydrogen bonding have been excluded.

**Figure 3:**
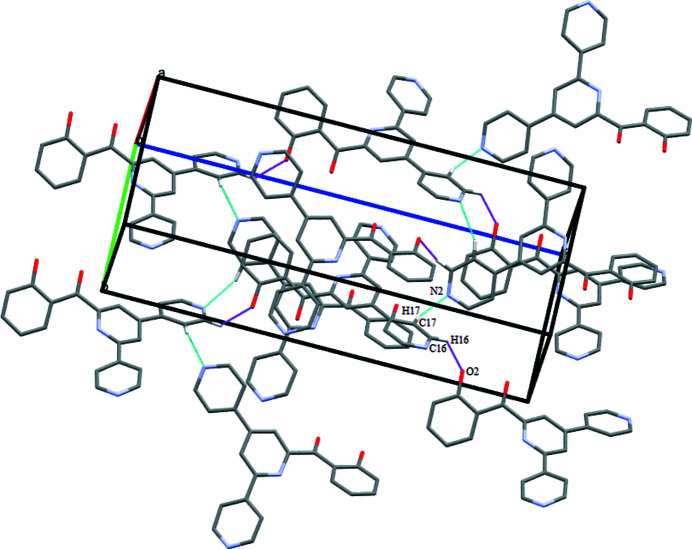
Unit-cell packing of the title compound showing inter­molecular C—H⋯N and C—H⋯O inter­actions as dotted lines. H atoms not involved in hydrogen bonding have been excluded.

**Figure 4:**
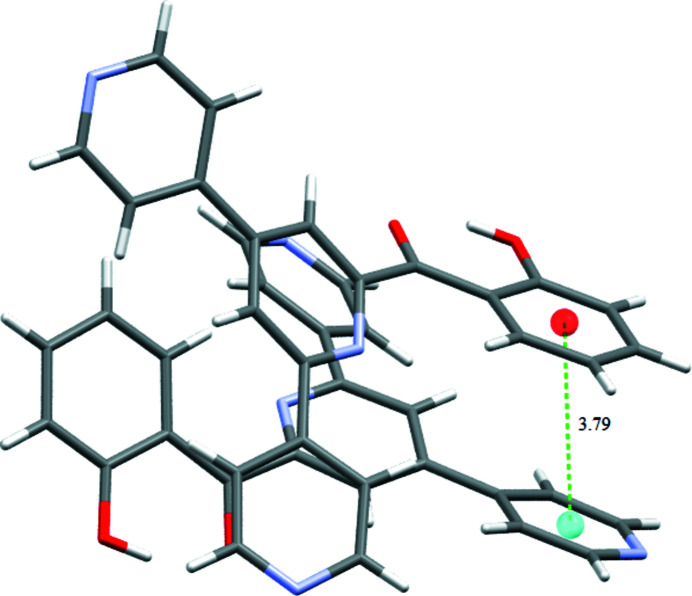
Unit-cell packing depicting the inter­molecular π–π stacking inter­actions as dotted lines.

The crystal packing features the C21—H21⋯O1 inter­action along the crystallographic *a* axis. The O1 atom of the phenol group is involves in both intra- and inter-mol­ecular hydrogen bonding (Table 1 ▸, Fig. 2 ▸). The crystal structure is further stabilized by the following inter­molecular inter­actions: C17—H17⋯N2^ii^ [symmetry code: (ii) −*x* + 1, *y* − 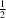 , −*z* + 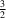 ], which connects the mol­ecules in a zigzag pattern, and C16—H16⋯O2^i^ [symmetry code: (i) −*x* + 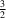 , −*y* + 1, *z* − 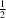 ], which forms mol­ecular chains along the crystallographic *b* and *a* axes, respectively (Fig. 3 ▸). In addition, a weak π–π stacking inter­action exhibiting a centroid–centroid distance of 3.7919 (15) Å between C7–C12 and C13–C17/N2 rings is observed (Fig. 4 ▸).

## Synthesis and crystallization

A mixture of 3-amino-4-hy­droxy­coumarin (10 mmol, 1.0 equiv.), 4-acetyl­pyridine (22 mmol, 2.2 equiv.) and a few drops of TEA (0.2 equiv.) in toluene (25 ml) was refluxed under nitro­gen for 4 h. The progress of the reaction was monitored by TLC. The reaction mixture was allowed to attain room temperature and the solvent was dried using a rotary evaporator. The crude product was purified by flash column chromatography to obtain a yellowish brown solid with 67% yield, *R*_f_ = 0.10 (5% DCM/MeOH), m.p. 190–192°C. The compound was recrystallized from ethanol solution. ^1^H NMR (CDCl_3_, 400 MHz) δ 12.13 (*s*, 1H), 8.84 (*d*, *J* = 6.4 Hz, 2H), 8.81 (*d*, *J* = 6.4 Hz, 2H), 8.25 (*dd*, *J* = 8.0, 1.2 Hz, 1H), 8.22 (*d*, *J* = 1.2 Hz, 1H), 8.19 (*d*, *J* = 1.2 Hz, 1H), 8.01 (*dd*, *J* = 4.8, 1.6 Hz, 2H), 7.66 (*dd*, *J* = 3.2, 1.6 Hz, 2H), 7.58 (*t*, *J* = 7.6 Hz, 1H), 7.12 (*d*, *J* = 8.4–0 Hz, 1H), 6.96 (*t*, *J* = 7.2 Hz, 1H); ^13^C NMR (CDCl_3_, 150 MHz) δ 196.7, 164.9, 156.6, 154.4, 150.9, 150.8, 150.7, 148.7, 144.8, 144.6, 137.1, 134.2, 122.3, 121.4, 121.1, 120.4, 118.9, 118.5.

## Refinement

Crystal data, data collection and structure refinement details are summarized in Table 2 ▸.

## Supplementary Material

Crystal structure: contains datablock(s) global, I. DOI: 10.1107/S2414314620008573/zq4040sup1.cif

Structure factors: contains datablock(s) I. DOI: 10.1107/S2414314620008573/zq4040Isup2.hkl

Supporting information file. DOI: 10.1107/S2414314620008573/zq4040Isup6.cml

Reaction Scheme. DOI: 10.1107/S2414314620008573/zq4040sup3.png

Proton-NMR. DOI: 10.1107/S2414314620008573/zq4040sup4.pdf

13C-NMR. DOI: 10.1107/S2414314620008573/zq4040sup5.pdf

CCDC reference: 2012289

Additional supporting information:  crystallographic information; 3D view; checkCIF report

## Figures and Tables

**Table 1:** Hydrogen-bond geometry (Å, °)

*D*—H⋯*A*	*D*—H	H⋯*A*	*D*⋯*A*	*D*—H⋯*A*
O2—H2′⋯O1	0.87 (4)	1.73 (4)	2.527 (3)	152 (3)
C16—H16⋯O2^i^	0.95	2.59	3.309 (3)	132
C17—H17⋯N2^ii^	0.95	2.49	3.344 (3)	150
C21—H21⋯O1^iii^	0.95	2.45	3.380 (3)	165

Symmetry codes: (i) 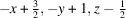 ; (ii) 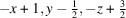 ; (iii) 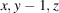 .

**Table 2:** Experimental details

Crystal data	
Chemical formula	C_22_H_15_N_3_O_2_
*M*_r_	353.37
Crystal system, space group	Orthorhombic, *P*2_1_2_1_2_1_
Temperature (K)	150
*a*, *b*, *c* (Å)	7.2994 (2), 9.6105 (3), 23.9296 (8)
*V* (Å^3^)	1678.68 (9)
*Z*	4
Radiation type	Mo *K*α
μ (mm^−1^)	0.09
Crystal size (mm)	0.48 × 0.25 × 0.18

Data collection	
Diffractometer	Bruker SMART APEX CCD
Absorption correction	Multi-scan (*SADABS*; Bruker, 1998 ▸)
*T*_min_, *T*_max_	0.879, 0.928
No. of measured, independent and observed [*I* > 2σ(*I*)] reflections	23873, 3416, 3003
*R*_int_	0.041
(sin θ/λ)_max_ (Å^−1^)	0.626

Refinement	
*R*[*F* ^2^ > 2σ(*F* ^2^)], *wR*(*F* ^2^), *S*	0.038, 0.088, 1.10
No. of reflections	3416
No. of parameters	248
H-atom treatment	H atoms treated by a mixture of independent and constrained refinement
Δρ_max_, Δρ_min_ (e Å^−3^)	0.18, −0.23

Computer programs: *SMART* and *SAINT* (Bruker, 1998 ▸), *SHELXS97* (Sheldrick, 2008 ▸), *SHELXL2018/3* (Sheldrick, 2015 ▸) and *ORTEP-3 for Windows* (Farrugia, 2012 ▸).

## full crystallographic data

### Crystal data

**Table:**

C_22_H_15_N_3_O_2_	*D*_x_ = 1.398 Mg m^−^^3^
*M**_r_* = 353.37	Mo *K*α radiation, λ = 0.71073 Å
Orthorhombic, *P*2_1_2_1_2_1_	Cell parameters from 9903 reflections
*a* = 7.2994 (2) Å	θ = 2.9–26.3°
*b* = 9.6105 (3) Å	µ = 0.09 mm^−^^1^
*c* = 23.9296 (8) Å	*T* = 150 K
*V* = 1678.68 (9) Å^3^	Parallelepiped, yellow
*Z* = 4	0.48 × 0.25 × 0.18 mm
*F*(000) = 736	

### Data collection

**Table:**

Bruker SMART APEX CCD diffractometer	3003 reflections with *I* > 2σ(*I*)
Radiation source: fine-focus sealed tube	*R*_int_ = 0.041
ω scans	θ_max_ = 26.4°, θ_min_ = 3.3°
Absorption correction: multi-scan (SADABS; Bruker, 1998)	*h* = −9→8
*T*_min_ = 0.879, *T*_max_ = 0.928	*k* = −12→11
23873 measured reflections	*l* = −29→29
3416 independent reflections	

### Refinement

**Table:**

Refinement on *F*^2^	Primary atom site location: structure-invariant direct methods
Least-squares matrix: full	Secondary atom site location: difference Fourier map
*R*[*F*^2^ > 2σ(*F*^2^)] = 0.038	Hydrogen site location: mixed
*wR*(*F*^2^) = 0.088	H atoms treated by a mixture of independent and constrained refinement
*S* = 1.10	*w* = 1/[σ^2^(*F*_o_^2^) + (0.0377*P*)^2^ + 0.4435*P*] where *P* = (*F*_o_^2^ + 2*F*_c_^2^)/3
3416 reflections	(Δ/σ)_max_ = 0.001
248 parameters	Δρ_max_ = 0.18 e Å^−^^3^
0 restraints	Δρ_min_ = −0.23 e Å^−^^3^

### Special details

**Table:**

Geometry. All esds (except the esd in the dihedral angle between two l.s. planes) are estimated using the full covariance matrix. The cell esds are taken into account individually in the estimation of esds in distances, angles and torsion angles; correlations between esds in cell parameters are only used when they are defined by crystal symmetry. An approximate (isotropic) treatment of cell esds is used for estimating esds involving l.s. planes.
Refinement. The hydroxy H atom was located in the Fourier maps and freyly refined, while all other H atoms were fixed geometrically and allow to ride on their parent carbon atoms, with a C—H distance of 0.95 Å and *U*_iso_(H) = 1.2*U*_eq_(C).

### Fractional atomic coordinates and isotropic or equivalent isotropic displacement parameters (Å^2^)

**Table:**

	*x*	*y*	*z*	*U*_iso_*/*U*_eq_	
O1	0.6864 (3)	0.49905 (18)	1.05784 (7)	0.0296 (4)	
O2	0.7035 (3)	0.49051 (18)	1.16325 (7)	0.0321 (4)	
H2'	0.706 (5)	0.522 (3)	1.1292 (14)	0.051 (10)*	
N1	0.6398 (3)	0.18406 (19)	0.98997 (7)	0.0195 (4)	
N2	0.3663 (3)	0.6191 (2)	0.76383 (9)	0.0348 (6)	
N3	0.8135 (4)	−0.2862 (2)	0.91678 (9)	0.0367 (6)	
C1	0.6004 (3)	0.3190 (2)	0.99800 (9)	0.0193 (5)	
C2	0.5477 (3)	0.4093 (2)	0.95579 (9)	0.0201 (5)	
H2	0.525464	0.504644	0.963740	0.024*	
C3	0.5278 (3)	0.3587 (2)	0.90164 (9)	0.0197 (5)	
C4	0.5690 (3)	0.2187 (2)	0.89279 (9)	0.0199 (5)	
H4	0.557492	0.179870	0.856446	0.024*	
C5	0.6269 (3)	0.1354 (2)	0.93715 (9)	0.0194 (5)	
C6	0.6289 (3)	0.3781 (2)	1.05576 (9)	0.0211 (5)	
C7	0.5894 (3)	0.2996 (2)	1.10698 (9)	0.0195 (5)	
C8	0.5071 (3)	0.1676 (2)	1.10724 (10)	0.0220 (5)	
H8	0.476367	0.124708	1.072717	0.026*	
C9	0.4699 (3)	0.0988 (3)	1.15652 (10)	0.0263 (5)	
H9	0.414255	0.009436	1.155914	0.032*	
C10	0.5147 (4)	0.1617 (3)	1.20729 (10)	0.0296 (6)	
H10	0.490145	0.114222	1.241306	0.036*	
C11	0.5937 (4)	0.2912 (3)	1.20868 (10)	0.0294 (6)	
H11	0.623645	0.332654	1.243546	0.035*	
C12	0.6301 (3)	0.3618 (2)	1.15932 (10)	0.0230 (5)	
C13	0.4716 (3)	0.4501 (2)	0.85454 (9)	0.0208 (5)	
C14	0.3732 (4)	0.5721 (3)	0.86268 (10)	0.0281 (6)	
H14	0.339665	0.600640	0.899286	0.034*	
C15	0.3244 (4)	0.6518 (3)	0.81667 (11)	0.0350 (6)	
H15	0.256962	0.734852	0.823080	0.042*	
C16	0.4630 (4)	0.5027 (3)	0.75683 (11)	0.0315 (6)	
H16	0.496579	0.477605	0.719817	0.038*	
C17	0.5177 (3)	0.4161 (3)	0.79977 (10)	0.0248 (5)	
H17	0.586121	0.334272	0.792006	0.030*	
C18	0.6840 (3)	−0.0113 (2)	0.92905 (9)	0.0205 (5)	
C19	0.7538 (3)	−0.0593 (3)	0.87856 (10)	0.0252 (5)	
H19	0.759270	0.000724	0.847051	0.030*	
C20	0.8152 (4)	−0.1948 (3)	0.87460 (11)	0.0333 (6)	
H20	0.861862	−0.225112	0.839599	0.040*	
C21	0.7440 (4)	−0.2392 (3)	0.96517 (11)	0.0321 (6)	
H21	0.739621	−0.301578	0.995929	0.039*	
C22	0.6785 (4)	−0.1058 (2)	0.97307 (10)	0.0250 (5)	
H22	0.630106	−0.078786	1.008290	0.030*	

### Atomic displacement parameters (Å^2^)

**Table:**

	*U*^11^	*U*^22^	*U*^33^	*U*^12^	*U*^13^	*U*^23^
O1	0.0424 (11)	0.0236 (9)	0.0228 (9)	−0.0076 (8)	−0.0032 (8)	0.0015 (7)
O2	0.0489 (12)	0.0263 (10)	0.0212 (9)	−0.0064 (8)	−0.0069 (8)	−0.0005 (8)
N1	0.0189 (10)	0.0212 (10)	0.0183 (10)	−0.0016 (8)	0.0001 (7)	0.0018 (8)
N2	0.0347 (13)	0.0338 (13)	0.0360 (13)	0.0014 (11)	−0.0033 (10)	0.0151 (10)
N3	0.0515 (15)	0.0219 (12)	0.0366 (13)	0.0032 (11)	−0.0013 (11)	−0.0022 (9)
C1	0.0161 (11)	0.0223 (12)	0.0195 (11)	−0.0022 (9)	0.0010 (9)	−0.0001 (9)
C2	0.0198 (11)	0.0175 (12)	0.0231 (12)	−0.0018 (9)	0.0003 (9)	−0.0004 (9)
C3	0.0155 (11)	0.0227 (12)	0.0208 (11)	−0.0013 (9)	−0.0003 (9)	0.0024 (9)
C4	0.0187 (11)	0.0230 (12)	0.0179 (11)	−0.0019 (9)	−0.0020 (9)	0.0001 (9)
C5	0.0178 (11)	0.0194 (12)	0.0211 (11)	−0.0033 (9)	0.0003 (9)	0.0017 (9)
C6	0.0202 (12)	0.0213 (12)	0.0217 (12)	0.0020 (10)	−0.0021 (9)	0.0004 (10)
C7	0.0187 (11)	0.0215 (12)	0.0183 (11)	0.0040 (9)	−0.0014 (9)	−0.0001 (9)
C8	0.0196 (11)	0.0247 (13)	0.0217 (11)	0.0013 (9)	0.0007 (9)	−0.0017 (10)
C9	0.0253 (12)	0.0242 (13)	0.0294 (13)	−0.0001 (10)	0.0057 (11)	0.0035 (10)
C10	0.0345 (14)	0.0326 (14)	0.0217 (12)	0.0038 (12)	0.0047 (11)	0.0075 (11)
C11	0.0375 (15)	0.0333 (14)	0.0175 (12)	0.0053 (12)	−0.0012 (10)	−0.0018 (11)
C12	0.0262 (12)	0.0211 (12)	0.0218 (12)	0.0030 (10)	−0.0012 (10)	−0.0008 (10)
C13	0.0183 (11)	0.0207 (11)	0.0232 (11)	−0.0019 (9)	−0.0035 (9)	0.0032 (10)
C14	0.0312 (13)	0.0259 (13)	0.0272 (13)	0.0026 (11)	−0.0017 (11)	0.0020 (10)
C15	0.0371 (15)	0.0257 (14)	0.0423 (16)	0.0063 (12)	−0.0035 (13)	0.0064 (12)
C16	0.0299 (14)	0.0412 (16)	0.0235 (13)	−0.0002 (12)	−0.0019 (11)	0.0080 (12)
C17	0.0243 (13)	0.0267 (14)	0.0233 (12)	0.0018 (10)	−0.0031 (10)	0.0010 (10)
C18	0.0195 (12)	0.0209 (12)	0.0209 (11)	−0.0032 (10)	−0.0040 (9)	−0.0004 (9)
C19	0.0304 (13)	0.0226 (12)	0.0226 (12)	−0.0020 (10)	−0.0026 (10)	0.0011 (10)
C20	0.0436 (15)	0.0279 (14)	0.0283 (13)	0.0019 (12)	0.0018 (12)	−0.0040 (11)
C21	0.0444 (17)	0.0220 (13)	0.0299 (13)	−0.0020 (12)	−0.0046 (12)	0.0047 (11)
C22	0.0315 (13)	0.0227 (13)	0.0208 (12)	−0.0013 (10)	−0.0006 (10)	0.0011 (9)

### Geometric parameters (Å, º)

**Table:**

O1—C6	1.237 (3)	C9—C10	1.396 (3)
O2—C12	1.351 (3)	C9—H9	0.9500
O2—H2'	0.87 (3)	C10—C11	1.372 (4)
N1—C1	1.342 (3)	C10—H10	0.9500
N1—C5	1.351 (3)	C11—C12	1.388 (3)
N2—C16	1.334 (3)	C11—H11	0.9500
N2—C15	1.338 (3)	C13—C14	1.389 (3)
N3—C20	1.338 (3)	C13—C17	1.392 (3)
N3—C21	1.343 (3)	C14—C15	1.388 (3)
C1—C2	1.386 (3)	C14—H14	0.9500
C1—C6	1.509 (3)	C15—H15	0.9500
C2—C3	1.392 (3)	C16—C17	1.381 (3)
C2—H2	0.9500	C16—H16	0.9500
C3—C4	1.395 (3)	C17—H17	0.9500
C3—C13	1.487 (3)	C18—C19	1.390 (3)
C4—C5	1.395 (3)	C18—C22	1.391 (3)
C4—H4	0.9500	C19—C20	1.380 (3)
C5—C18	1.484 (3)	C19—H19	0.9500
C6—C7	1.467 (3)	C20—H20	0.9500
C7—C8	1.404 (3)	C21—C22	1.381 (4)
C7—C12	1.419 (3)	C21—H21	0.9500
C8—C9	1.379 (3)	C22—H22	0.9500
C8—H8	0.9500		
			
C12—O2—H2'	105 (2)	C10—C11—H11	119.9
C1—N1—C5	116.96 (19)	C12—C11—H11	119.9
C16—N2—C15	115.9 (2)	O2—C12—C11	117.7 (2)
C20—N3—C21	115.7 (2)	O2—C12—C7	122.0 (2)
N1—C1—C2	124.1 (2)	C11—C12—C7	120.3 (2)
N1—C1—C6	117.72 (19)	C14—C13—C17	117.1 (2)
C2—C1—C6	118.1 (2)	C14—C13—C3	122.4 (2)
C1—C2—C3	119.3 (2)	C17—C13—C3	120.6 (2)
C1—C2—H2	120.4	C15—C14—C13	119.2 (2)
C3—C2—H2	120.4	C15—C14—H14	120.4
C2—C3—C4	117.1 (2)	C13—C14—H14	120.4
C2—C3—C13	121.9 (2)	N2—C15—C14	124.1 (2)
C4—C3—C13	120.9 (2)	N2—C15—H15	117.9
C3—C4—C5	120.2 (2)	C14—C15—H15	117.9
C3—C4—H4	119.9	N2—C16—C17	124.4 (2)
C5—C4—H4	119.9	N2—C16—H16	117.8
N1—C5—C4	122.3 (2)	C17—C16—H16	117.8
N1—C5—C18	115.6 (2)	C16—C17—C13	119.3 (2)
C4—C5—C18	122.1 (2)	C16—C17—H17	120.3
O1—C6—C7	121.0 (2)	C13—C17—H17	120.3
O1—C6—C1	115.9 (2)	C19—C18—C22	116.9 (2)
C7—C6—C1	123.0 (2)	C19—C18—C5	122.1 (2)
C8—C7—C12	117.8 (2)	C22—C18—C5	120.9 (2)
C8—C7—C6	123.5 (2)	C20—C19—C18	119.4 (2)
C12—C7—C6	118.7 (2)	C20—C19—H19	120.3
C9—C8—C7	121.4 (2)	C18—C19—H19	120.3
C9—C8—H8	119.3	N3—C20—C19	124.4 (2)
C7—C8—H8	119.3	N3—C20—H20	117.8
C8—C9—C10	119.4 (2)	C19—C20—H20	117.8
C8—C9—H9	120.3	N3—C21—C22	124.2 (2)
C10—C9—H9	120.3	N3—C21—H21	117.9
C11—C10—C9	120.8 (2)	C22—C21—H21	117.9
C11—C10—H10	119.6	C21—C22—C18	119.5 (2)
C9—C10—H10	119.6	C21—C22—H22	120.3
C10—C11—C12	120.2 (2)	C18—C22—H22	120.3
			
C5—N1—C1—C2	0.2 (3)	C6—C7—C12—O2	0.1 (3)
C5—N1—C1—C6	−175.1 (2)	C8—C7—C12—C11	1.9 (3)
N1—C1—C2—C3	2.1 (3)	C6—C7—C12—C11	179.6 (2)
C6—C1—C2—C3	177.4 (2)	C2—C3—C13—C14	−24.2 (3)
C1—C2—C3—C4	−2.1 (3)	C4—C3—C13—C14	158.0 (2)
C1—C2—C3—C13	180.0 (2)	C2—C3—C13—C17	155.4 (2)
C2—C3—C4—C5	0.1 (3)	C4—C3—C13—C17	−22.4 (3)
C13—C3—C4—C5	178.0 (2)	C17—C13—C14—C15	0.8 (4)
C1—N1—C5—C4	−2.3 (3)	C3—C13—C14—C15	−179.6 (2)
C1—N1—C5—C18	175.8 (2)	C16—N2—C15—C14	−0.8 (4)
C3—C4—C5—N1	2.2 (3)	C13—C14—C15—N2	−0.1 (4)
C3—C4—C5—C18	−175.8 (2)	C15—N2—C16—C17	1.0 (4)
N1—C1—C6—O1	143.3 (2)	N2—C16—C17—C13	−0.3 (4)
C2—C1—C6—O1	−32.3 (3)	C14—C13—C17—C16	−0.7 (3)
N1—C1—C6—C7	−37.6 (3)	C3—C13—C17—C16	179.7 (2)
C2—C1—C6—C7	146.9 (2)	N1—C5—C18—C19	−151.7 (2)
O1—C6—C7—C8	173.4 (2)	C4—C5—C18—C19	26.4 (3)
C1—C6—C7—C8	−5.7 (3)	N1—C5—C18—C22	24.9 (3)
O1—C6—C7—C12	−4.2 (3)	C4—C5—C18—C22	−157.0 (2)
C1—C6—C7—C12	176.7 (2)	C22—C18—C19—C20	−0.7 (4)
C12—C7—C8—C9	−1.3 (3)	C5—C18—C19—C20	176.0 (2)
C6—C7—C8—C9	−178.9 (2)	C21—N3—C20—C19	0.9 (4)
C7—C8—C9—C10	0.1 (3)	C18—C19—C20—N3	−0.3 (4)
C8—C9—C10—C11	0.5 (4)	C20—N3—C21—C22	−0.5 (4)
C9—C10—C11—C12	0.1 (4)	N3—C21—C22—C18	−0.4 (4)
C10—C11—C12—O2	178.2 (2)	C19—C18—C22—C21	1.1 (4)
C10—C11—C12—C7	−1.3 (4)	C5—C18—C22—C21	−175.7 (2)
C8—C7—C12—O2	−177.7 (2)		

### Hydrogen-bond geometry (Å, º)

**Table:**

*D*—H···*A*	*D*—H	H···*A*	*D*···*A*	*D*—H···*A*
O2—H2′···O1	0.87 (4)	1.73 (4)	2.527 (3)	152 (3)
C16—H16···O2^i^	0.95	2.59	3.309 (3)	132
C17—H17···N2^ii^	0.95	2.49	3.344 (3)	150
C21—H21···O1^iii^	0.95	2.45	3.380 (3)	165

Symmetry codes: (i) −*x*+3/2, −*y*+1, *z*−1/2; (ii) −*x*+1, *y*−1/2, −*z*+3/2; (iii) *x*, *y*−1, *z*.
