# Supplementary figures and images for: (2-Hy­droxy­phen­yl)(4,2′:4′,4′′-terpyridin-6′-yl)methanone
Source: IUCrdata. 2020 Jul 10;5(Pt 7):x200857. doi: 10.1107/S2414314620008573 (PMC9462256; doi:10.1107/S2414314620008573)

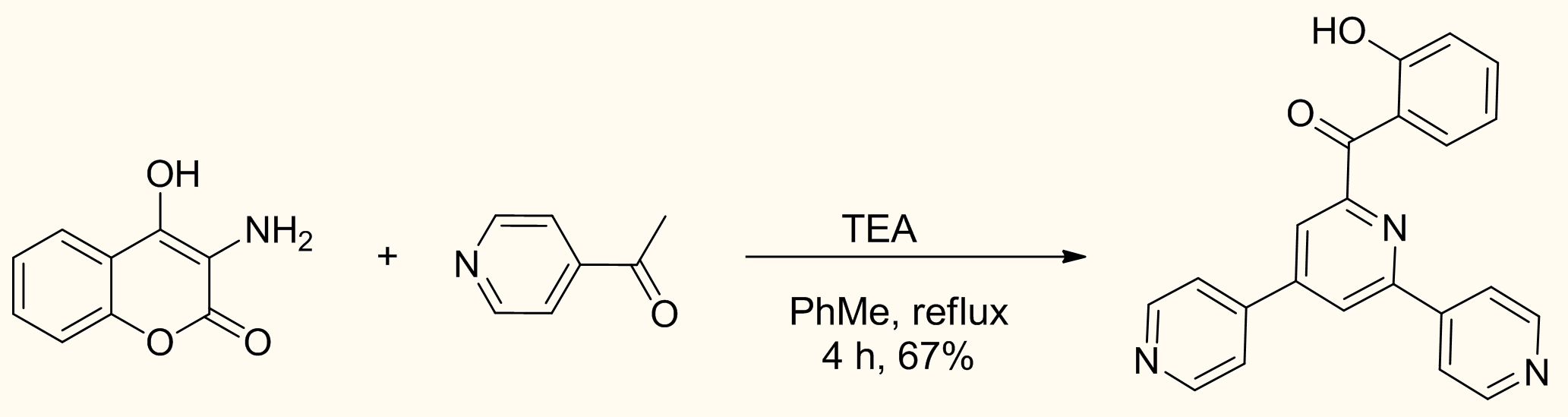

Supplement: Supplementary file 4 [file x-05-x200857-sup3.png]

DYY-BMK-5-913-A

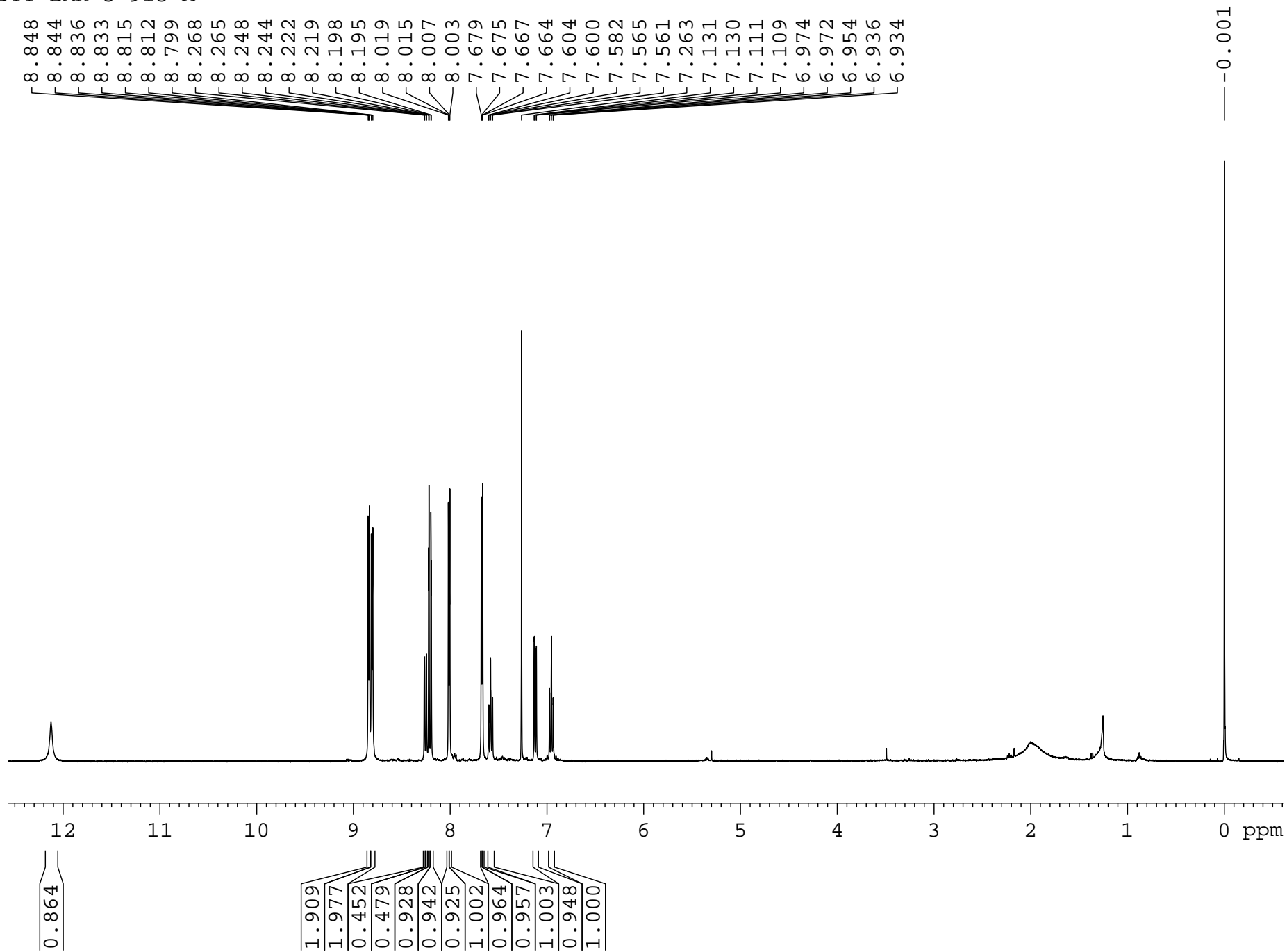

DYY-BMK-5-913-A

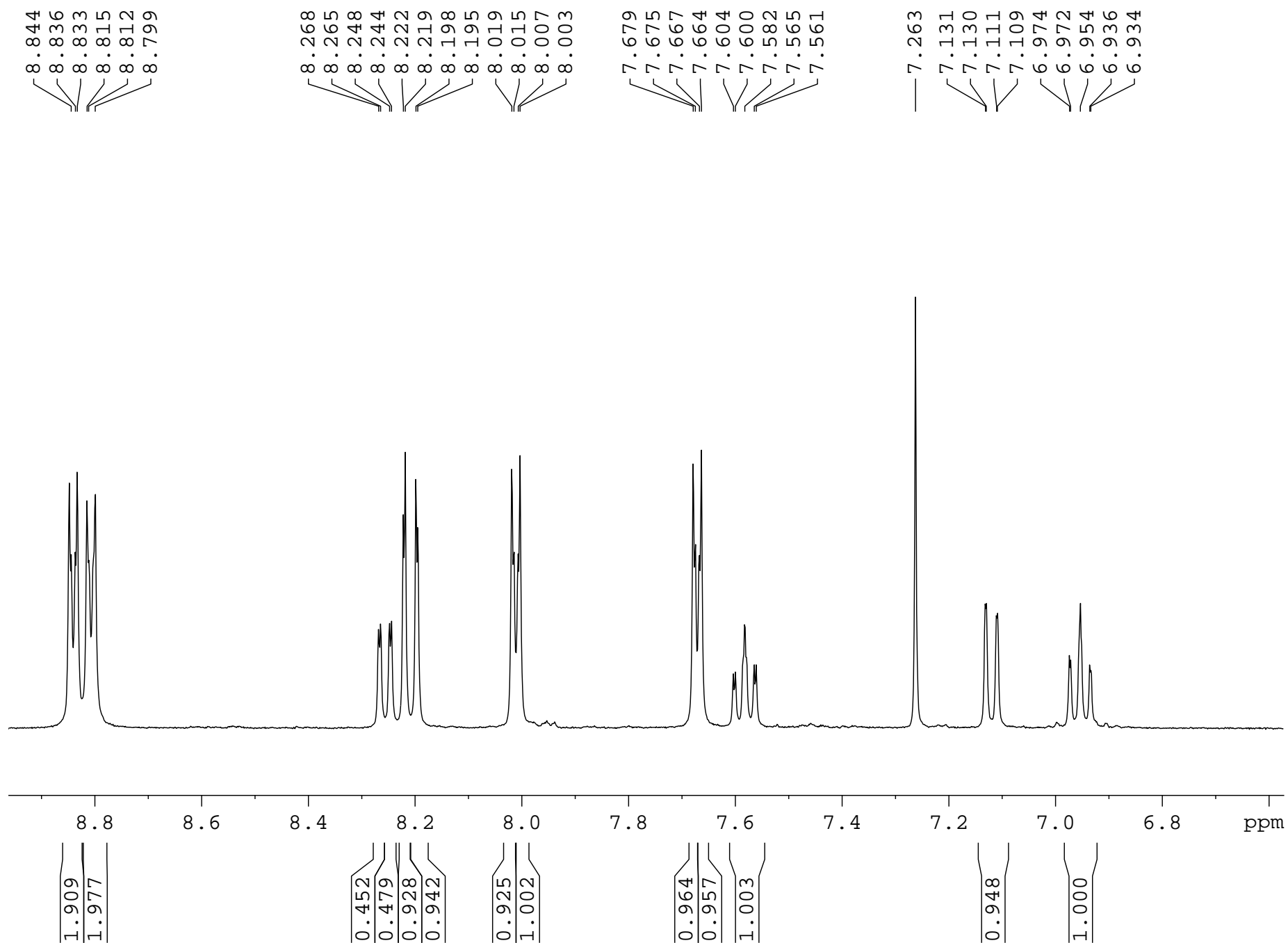

Supplement: Supplementary file 5 [file x-05-x200857-sup4.pdf]

DYY-BMK-5-913C

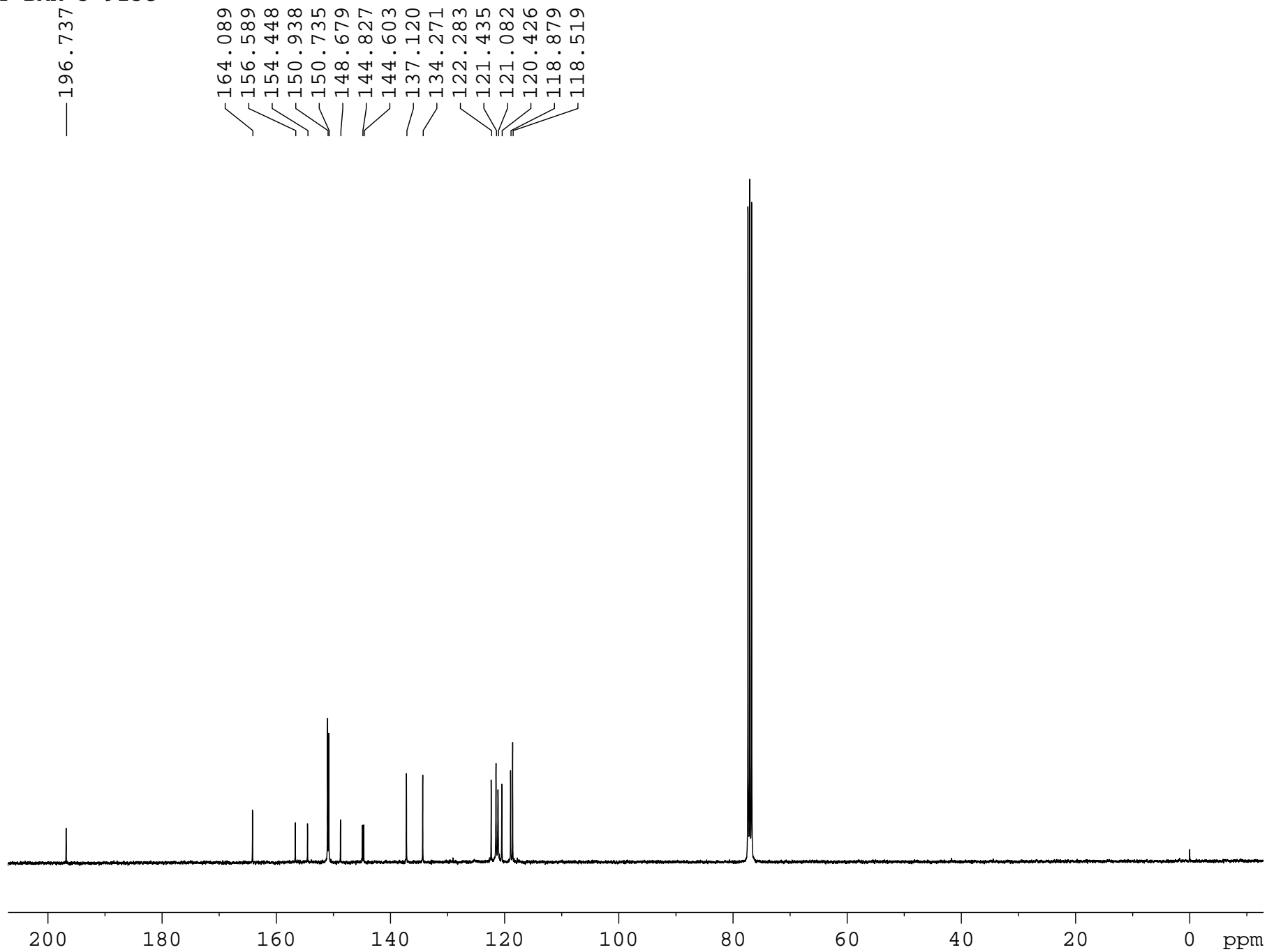

Supplement: Supplementary file 6 [file x-05-x200857-sup5.pdf]
